# Ultrathin permselective membranes: the latent way for efficient gas separation

**DOI:** 10.1039/d0ra02254c

**Published:** 2020-03-27

**Authors:** Roberto Castro-Muñoz, Kumar Varoon Agrawal, Joaquín Coronas

**Affiliations:** Tecnologico de Monterrey, Campus Toluca Avenida Eduardo Monroy Cárdenas 2000 San Antonio Buenavista 50110 Toluca de Lerdo Mexico food.biotechnology88@gmail.com castromr@tec.mx; Institute of Chemical Sciences and Engineering (ISIC), École Polytechnique Fédérale de Lausanne Sion Switzerland kumar.agrawal@epfl.ch; Chemical and Environmental Engineering Department, Instituto de Nanociencia de Aragón (INA), Instituto de Ciencia de Materiales de Aragón (ICMA), Universidad de Zaragoza-CSIC 50018 Zaragoza Spain coronas@unizar.es

## Abstract

Membrane gas separation has attracted the attention of chemical engineers for the selective separation of gases. Among the different types of membranes used, ultrathin membranes are recognized to break the trade-off between selectivity and permeance to provide ultimate separation. Such success has been associated with the ultrathin nature of the selective layer as well as their defect-free structure. These membrane features can be obtained from specific membrane preparation procedures used, in which the intrinsic properties of different nanostructured materials (*e.g.*, polymers, zeolites, covalent–organic frameworks, metal–organic frameworks, and graphene and its derivatives) also play a crucial role. It is likely that such a concept of membranes will be explored in the coming years. Therefore, the goal of this review study is to give the latest insights into the use of ultrathin selective barriers, highlighting and describing the primary membrane preparation protocols applied, such as atomic layer deposition, *in situ* crystal formation, interfacial polymerization, Langmuir–Blodgett technique, facile filtration process, and gutter layer formation, to mention just a few. For this, the most recent approaches are addressed, with particular emphasis on the most relevant results in separating gas molecules. A brief overview of the fundamentals for the application of the techniques is given. Finally, by reviewing the ongoing development works, the concluding remarks and future trends are also provided.

## Abstract 2

This review focuses on the application of ultrathin membranes for gas separation, describing the membrane preparation protocols applied. This includes atomic layer deposition, *in situ* crystal formation, interfacial polymerization and Langmuir–Blodgett technique.

## Graphical abstract

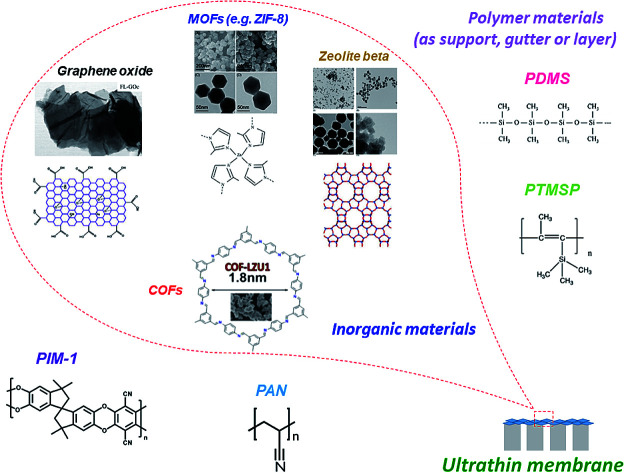

## 1. Introduction: the overview of new membrane concepts

**Fig. 1:**
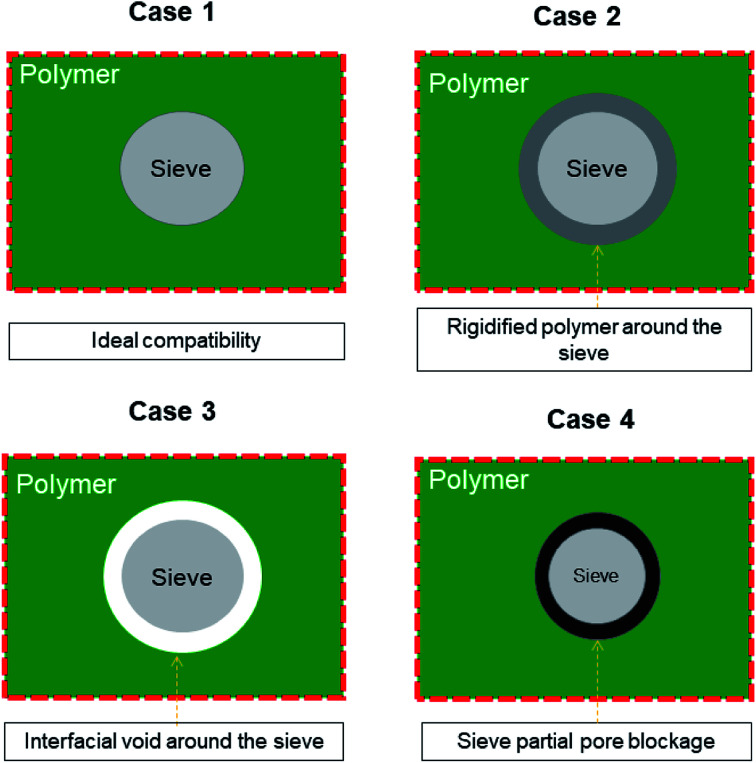
General representation of different structures at the polymer–filler interface region in MMMs.

Membrane-based technologies are attracting considerable attention for different types of approaches in the field of chemical engineering. In particular, membrane gas separation (GS) has been recognized since decades ago for its ability in separating gas mixtures of diverse molecules, such as organic (CH_4_, C_2_H_4_, C_2_H_6_, C_3_H_6_, C_3_H_8_, C_4_H_10_, *etc.*) and inorganic gases (CO_2_, H_2_, CO, N_2_, SF_6_, O_2_, He, and Ar).^1–3^ Importantly, Baker^4^ has pointed out that the use of GS industrially comprises the separation of non-condensable gases, including nitrogen (N_2_) from air, carbon dioxide (CO_2_) from methane (CH_4_), and hydrogen (H_2_) from N_2_, argon, or CH_4_. The benefit of using this membrane process is that it has low-energy requirements and shows high selectivity, efficiency, and feasibility in terms of scale-up.^5,6^ It is important to highlight Baker's idea that GS applications require the development of new membranes and membrane processes.^4^ To date, several polymer-based membranes have been developed in the field; however, highly selective polymers do not demonstrate high permeation rates and highly permeable polymers are not selective enough.^7,8^ These perm-selectivity limitations do not allow particular polymer membranes (*e.g.*, polyimides, poly(trimethylsilylpropyne), Teflon, polysulfone, cellulose acetate, and PDMS) to overcome the so-called Robeson trade-off, which was established to represent the relationship between permeability and selectivity towards specific gas pairs.^7–9^ Even if some pristine polymer membranes have shifted their performance (such as PIMs, thermally reduced polymers, Nafion®, Hyflon®, Viton®, Cytop™, and Teflon®AF) close to the upper bound relationship, their slope of the upper bound relationship has remained reasonably constant.^8,10^ Thereby, starting from the known separation features of the pristine polymers, several design concepts of new membranes have been developed, including cross-linked, polymer blending, annealed, composite, asymmetric and mixed matrix membranes (MMMs),^11^ and MMMs based on the copolymerization of organic macrocyclic molecules and microporous polymers.^12–17^ Among these types of membranes, the research community is looking for those involving nanostructured (inorganic, organic, or hybrid) materials, which once combined with polymer materials tend to result in a synergistic performance by combining the strengths of organic and inorganic materials. Nowadays, MMMs are likely the most explored membranes, often as a proof of concept for new porous fillers. In any event, the nanostructured materials used as fillers are contributing to the reduction in the drawbacks of polymer-based membranes, such as aging, plasticization phenomenon, and stability (*e.g.*, physical, chemical, and thermal).^18–21^ However, the unsuitable merging, including poor compatibility at the interface as well as membrane preparation protocol, make the filler–polymer membranes show specific defects (see Fig. 1), *e.g.*, new non-selective pathways for gas transport (case 3), which lead to an increase in the permeability but compromise on the selective properties.^22^ Of course, a good interface morphology (case 1) guarantees enhanced gas transport across the membranes, while other less favorable situations (cases 2 and 4) may compromise the expected increase in the performance of MMMs.

**Fig. 2:**
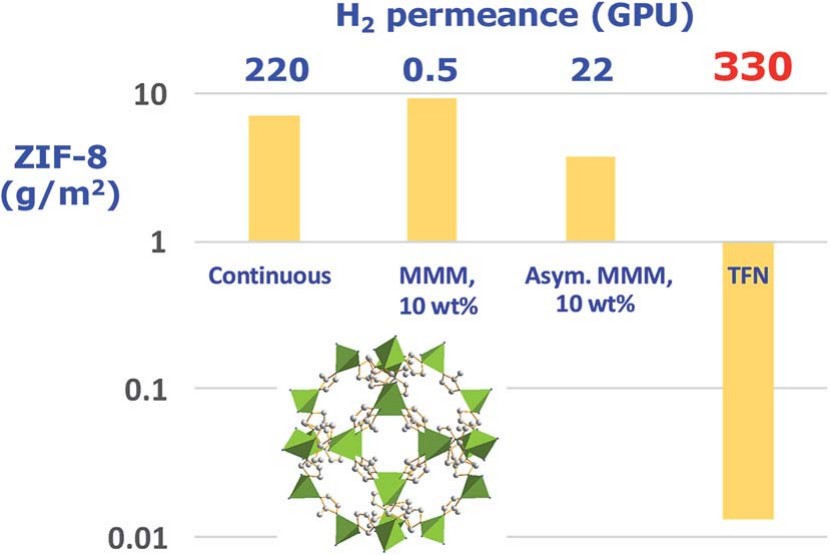
Amount of MOF ZIF-8 (ZIF-8 structure with the ZnN_4_ tetrahedra in green and carbon atoms from ligand molecules in grey. This structure was built with Diamond 3.2 using the corresponding crystallographic data,^23^ ref code: VELVOY01, CCDC: 602542) used in different types of gas selective membranes with their corresponding H_2_ permeance values. The thicknesses for continuous, MMMs (mixed matrix membrane), asymmetric MMMs, and TFN (thin film nanocomposite) membranes were 5 μm,^24^ 106 μm,^25^ 1 μm,^26^ and 100 nm (skin layer),^27^ respectively.

Compared with MMMs, ultrathin inorganic membranes from the corresponding nanosheets have been recognized as ideal candidates for obtaining superior performing membranes (see Fig. 2) and, importantly, using less amount of inorganic nanomaterials, which is in fact one of the main advantages of this type of membrane (see Table 1).

**Table 1:** Main advantages and drawbacks of ultrathin membranes compared to those of traditional ones

Advantages	Drawbacks
➢ Attractive separation performance	➢ Specific devices for membrane preparation
➢ Higher productivity (high permeances)	➢ Hydrothermal stability issues on single-layered layers based on MOFs
➢ Low amount of selective membrane-material	➢ Challenging spatial distribution
➢ Defect-free structure	➢ Physical aging and plasticization in the polymer support and polymer-based layers
➢ Less membrane area needed for a given separation	➢ Limited preparation procedures at a large scale
➢ Controlled grain size and thickness	➢ Separation properties depending on precursors and ligands
➢ Controllable tuning of the membrane pore size	➢ Hydrothermal stability of ligand–metal bonds
➢ Stable mechanical properties (*e.g.*, graphene-based membranes) for potential large-scale applications	
➢ Atomically thin membranes	
➢ Materials, fabrication, and energy cost savings	

This is due to the fact that they can be synthesized with a defect-free morphology, obtained by means of specific procedures applied for their preparation, including atomic layer deposition (ALD), solvothermal crystallization, interfacial crystallization, electrophoretic deposition (ED), chemical vapor deposition (CVD), Langmuir–Blodgett (LB) deposition, facile filtration process, and gutter layer formation.^28,29^ The use of thin membranes of nanostructured materials, dealing with the concept of zeolite films, was realized in 1989 with the synthesis of a hydrophilic zeolite on top of a porous glass support, which was able to dehydrate an organic solvent.^30^ The first zeolite membranes had thicknesses of about several tens of microns; for example, Geus *et al.*^31^ reported a *ca.* 50 μm thick silicalite-1 membrane in the year 1992. One of the most relevant publications on the synthesis of zeolite membranes, from the point of view of zeolite layer thickness, was by Hedlund *et al.*^32^ In the year 2002, they produced a 500 nm thick silicalite-1 membrane on a commercially available and technologically suitable alpha-alumina tube with an N_2_ permeance of almost 40 000 GPU (gas permeation unit, 1 GPU being 3.35 × 10^−10^ mol m^−2^ s^−1^ Pa^−1^), which is useful for xylene isomer separation. In the year 2015, 100 nm thick silicalite-1 membranes were reported by Tsapatsis *et al.* with *p*-xylene permeance exceeding 1000 GPU and with the *p*-xylene/*o*-xylene separation factor reaching 1000.^33^ The development of unit cell-thick, highly crystalline zeolite nanosheets in the last decade is likely to allow the synthesis of thin zeolite membranes by a simple assembly in the near future.

It is important to point out that industrialized state-of-the-art technologies were used to fabricate reverse osmosis and nanofiltration membranes since the end seventies^34^ based on the interfacial polymerization (IP) of aromatic polyamides producing the so-called thin-film composite membranes (*i.e.*, TFC and TFN membranes when incorporating fillers), which have endured two new evolutions. First, they have been recently applied for gas separation with good performance in H_2_/CO_2_ separation with H_2_ permeance in the range of 330–350 GPU.^27,35^ TFC membranes have controlling skin layers in the range of *ca.* 50 nm thickness and by special means, they can go down to *ca.* 10 nm,^36^ which explains their high permeances. Second, they have inspired the preparation of ultrathin films of porous coordination polymers by the same interfacial polymerization synthetic approach.^37^

**Fig. 3:**
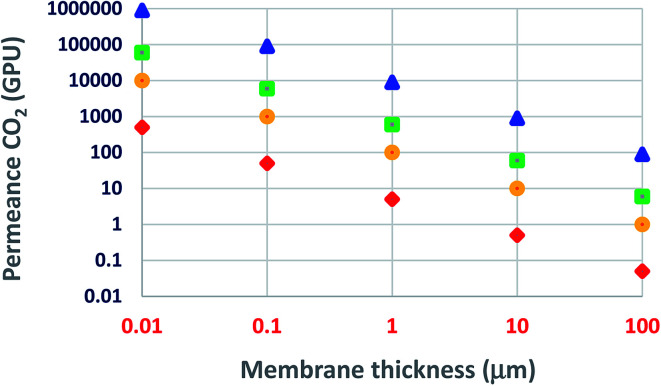
CO_2_ permeance as a function of membrane thickness: triangle, PIM-1; square, 6FDA-DAM; circle, Pebax® 1657; rhombus, polysulfone.^29,41–43^

Today, the use of ultrathin membranes provides some more advantages: (i) relatively small amounts of selective-layer materials, *e.g.*, 1 m^2^ of a 100 nm dense and continuous layer of ZIF-8 (density 0.95 g cm^−3^), ZSM-5 (1.8 g cm^−3^), or graphite (2.2 g cm^−3^) would require 95, 180, or 220 mg of the material coated on top of the porous structures, representing a significant savings in material costs; (ii) it leads to an optimized membrane material and morphology in each layer, (iii) minimal limitations on the mechanical properties and processability of membrane materials as long as they can be formed or deposited as a thin layer on the top.^38,39^ As the membrane thickness decreases, both the membrane cost and permeance are favored. Permeability (the typical Barrer units, 1 Barrer = 10^−10^ cm^3^ (STP) cm cm^−2^ s^−1^ cm_Hg_^−1^ = 3.35 × 10^−16^ mol mm^−2^ s^−1^ Pa^−1^) is the intrinsic transport parameter of a given membrane material. Thus, as Fig. 3 illustrates, for the same membrane material (*e.g.*, polysulfone, Pebax® 1657, polyimide 6FDA-DAM, or PIM-1), permeance increases as the membrane thickness decreases (when the membrane thickness is 1 μm, a permeability of 1 Barrer corresponds to a permeance of 1 GPU). Fig. 3 suggests that even a slightly permeable material such as polysulfone, maintaining its selectivity properties, could become a technologically attractive material when prepared as an ultrathin membrane. Herein, the challenge is to choose the right preparation methodology able to produce defect-free ultrathin membranes. It is essential to mention that at least in some cases, the permeability may decrease when one reduces the membrane thickness, for example, PDMS permeability decreased 100-fold when the thickness was reduced from 1 mm to 200 nm.^40^

It still remains a big challenge to overcome such trade-off issues to reach high permeance and selectivity at the same time, *i.e.*, to fabricate sub-1 μm thick membranes (even sub-100 nm) by using selective organic and inorganic materials^44^ such as polymers, zeolites, covalent–organic frameworks (COFs), metal–organic frameworks (MOFs), graphene, and carbon-based materials.^38^ Therefore, the goal of this review is to provide the latest insights into using ultrathin selective barriers, highlighting and describing the primary membrane preparation protocols used. Particular attention has been paid to the most relevant results in separating gas molecules. In addition, a brief overview of the fundamentals for the application of the techniques is given. Finally, by reviewing the ongoing development works, the concluding remarks and future perspectives are also addressed.

## 2. Fundamentals in membrane gas separation

In membrane gas separation, the membrane is certainly the primary tool for the separation of different gas molecules. To date, different gas transport mechanisms have been used to describe gas transport and thus, the separation mechanism in polymeric membranes, such as solution–diffusion transport, Knudsen-diffusion transport, surface diffusion, capillary condensation, viscous (Poiseuille) flow, and molecular sieving. Such mechanisms occur in membranes according to their structure (*i.e.*, porous or non-porous). In the case of nanoporous membranes, several transport mechanisms can manifest at the same time. For example, activated transport (molecular sieving) from the nanopores but Knudsen-diffusion from the grain boundary defects and viscous transport from the pinholes. On the other hand, in non-porous membranes, so-called dense membranes, the mechanism is mainly governed by solution–diffusion transport.^45^ In general, mass transfer across a dense membrane involves three main steps: (i) adsorption of the gas molecules from the mixture to the membrane on the basis of its chemical affinity, (ii) diffusion of the gas molecules through the membrane as a result of the chemical potential (*μ*_i_) and driving force, and (iii) desorption of the gas molecules at the permeate side of the membrane.^46^ The permeability (*P*) depends on the diffusivity (*D*) and solubility (*S*) of the transported molecules,^45^ as described by eqn (1). *S* is a thermodynamic parameter that provides insight into the amount of penetrant adsorbed by the membrane under equilibrium conditions, whereas *D* is a kinetic parameter that comprises the transport rate of the permeating molecules through the membrane.^47^ The two parameters can be denoted by the diffusion (*D*) and sorption (*S*) coefficients, respectively, and their product gives rise to permeability:

1 *P* = *DS*

where *P* is the permeability coefficient (cm^3^ (STP) cm cm^−2^ s^−1^ cm_Hg_^−1^), in which Barrer (10^−10^ cm^3^ (STP) cm cm^−2^ s^−1^ cm_Hg_^−1^) is the common unit. When the thickness of the active layer is not known, gas flows through the membrane can be determined by the gas permeation unit (GPU), which is expressed as 10^−6^ cm^3^ (STP) cm^−2^ s^−1^ cm_Hg_^−1^ (see above for alternative IS units). To determine the selectivity of a membrane, the ideal selectivity (*α*) can be determined as a relationship of the permeability of one gas (A) over that of the other gas (B), as shown in eqn (2). Moreover, it can be defined as the ratio between the diffusivity coefficients, known as “diffusion selectivity”, and the ratio between sorption coefficients, named as “solubility selectivity”.2
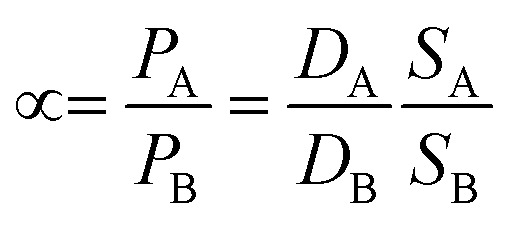


## 3. Preparation techniques of ultrathin organic/inorganic membranes: potential candidates for highly selective and permeable transport of gases

### 3.1 Advances in polymerization of ultrathin films

**Fig. 4:**
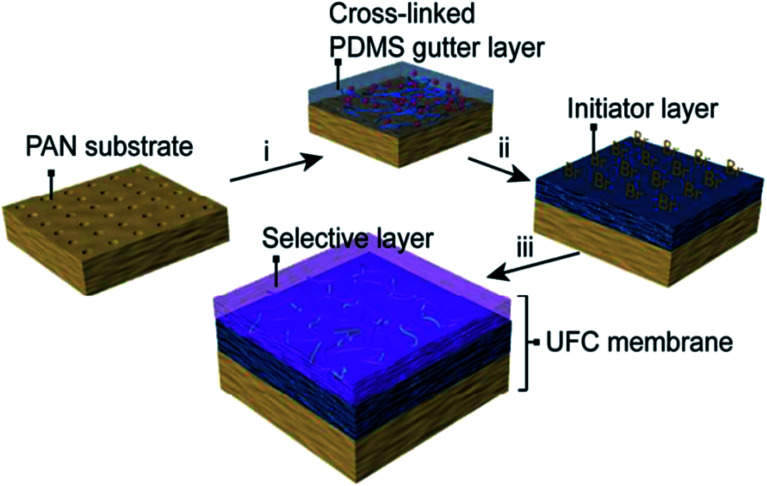
Schematic depiction of the ultra-TFC membranes developed by Fu *et al.*^51^

Today, the fabrication of an ultrathin selective layer onto a strong porous support is likely the most sought approach to develop next-generation high-performance membranes. In such an approach, the lamination of the layer generally employs the attachment of a previously prepared thin-film on top of a porous membrane support. *Plasma polymerization* protocol is a typical procedure that leads to the preparation of thin-film membranes,^48,49^ in which plasma polymers are coated on porous materials. Plasma polymerization is typically carried out within a vacuum system using helium or argon inert gases that induce plasma formation and, therefore, polymerization. This procedure possesses the advantage of forming an ultrathin defect-free film with thickness reaching a few nanometers, which is difficult to achieve by the conventional coating methods.^38^ On the contrary, such a technique finds its main drawback in the presence of many different reactive species (electrons, ions, radicals), which gives the possibility of multiple interactions of species. This makes it difficult to define the right chemical structure of the surface after exposure to a plasma.^38^ Fluorocarbon-based membranes have been prepared by such membrane preparation methods, which displayed a greater molecular sieving effect than those prepared by solution–diffusion separation.^50^ Recently, Fu *et al.*^51^ developed an ultrathin (∼100 nm) membrane with uniform thickness, which was able to facilitate high CO_2_ transport (*i.e.*, CO_2_ permeance = 1260 GPU and CO_2_/N_2_ selectivity = 43). This breakthrough was achieved by continuous assembly technology *via atom transfer radical polymerization* (ATRP) for defect-free thin-film nanocomposites (TFC) based on cross-linked PDMS ultrathin dense layer (*ca.* 40 nm). Such an ultrathin selective layer was made of PEG-based cross-linkers polymerized on top of a functionalized PDMS layer with double function of polymerization initiator and gutter layer onto a microporous PAN support (see Fig. 4).

Similarly, the preparation of a defect-free TFC membrane with a particular design has been documented by Xie *et al.*^52^ An ultrathin selective layer of about 30 nm was polymerized on a rough micro-scale MOF gutter layer. The polymer-on-MOF architecture provided impressive gas separation performance as well, for *e.g.*, CO_2_ permeance = 3000 GPU and CO_2_/N_2_ selectivity = 34. These results were obtained due to the fact that the MOF layer was about 400 times more permeable than the PDMS layer. In particular, NH_2_-MIL-53(Al) was the MOF used as it possesses a number of amino groups that can be functionalized with an atom-transfer radical polymerization initiator and thus provides the ability to form a continuous porous layer. Importantly, MOFs are a category of crystalline porous materials based on metal ions or clusters interconnected by organic ligands *via* coordination bonds to form one-, two-, or three-dimensional periodic networks.^13,53^ According to the metal properties (*e.g.*, polarizability, metal oxidation state, and ionic radius), the metal–ligand bond strength tends to vary.^54^ The synthetic route for forming MOF-based membranes on porous substrates is vastly different from that of MOF films on dense substrates. MOF membranes can be fabricated using various methods, including hydro/solvothermal synthesis, interfacial growth, CVD, ALD, and ED.^55,56^ The use of MOFs allows one to prepare ultrathin hybrid organic–inorganic selective membranes, for *e.g.*, using [Zn_2_(benzimidazole)_3_(OH)(H_2_O)]_*n*_ (hereafter abbreviated as Zn_2_(Bim)_3_) nanosheets.^39^ Herein, a modified soft *physical exfoliation method* was used to partially disintegrate a lamellar amphiprotic MOF into nanosheets. Afterwards, sub-10 nm-thick ultrathin membranes were successfully fabricated showing a suitable H_2_/CO_2_ separation performance, with a separation factor of 166 and with H_2_ permeance of up to 8 × 10^−7^ mol m^−2^ s^−1^ Pa^−1^ at elevated testing temperatures (200 °C), which is attributed to the size exclusion effect. In theory, H_2_ molecules (∼0.289 nm) can tightly pass through the apertures (∼0.29 nm) of the Zn_2_(Bim)_3_ nanosheets, while CO_2_ molecules (∼0.33 nm) and H_2_ can pass through the interlayer galleries. In fact, such molecular sieving mechanism was further proved by single permeation experiments through the nanosheet membranes; the experiments revealed a clear cut-off between H_2_ and other gas molecules having larger molecular diameters than the crystallographic pore size corresponding to Zn_2_(Bim)_3_ nanosheets.^39^

### 3.2 *In situ* growth and layer-by-layer (LBL) assembly

**Fig. 5:**
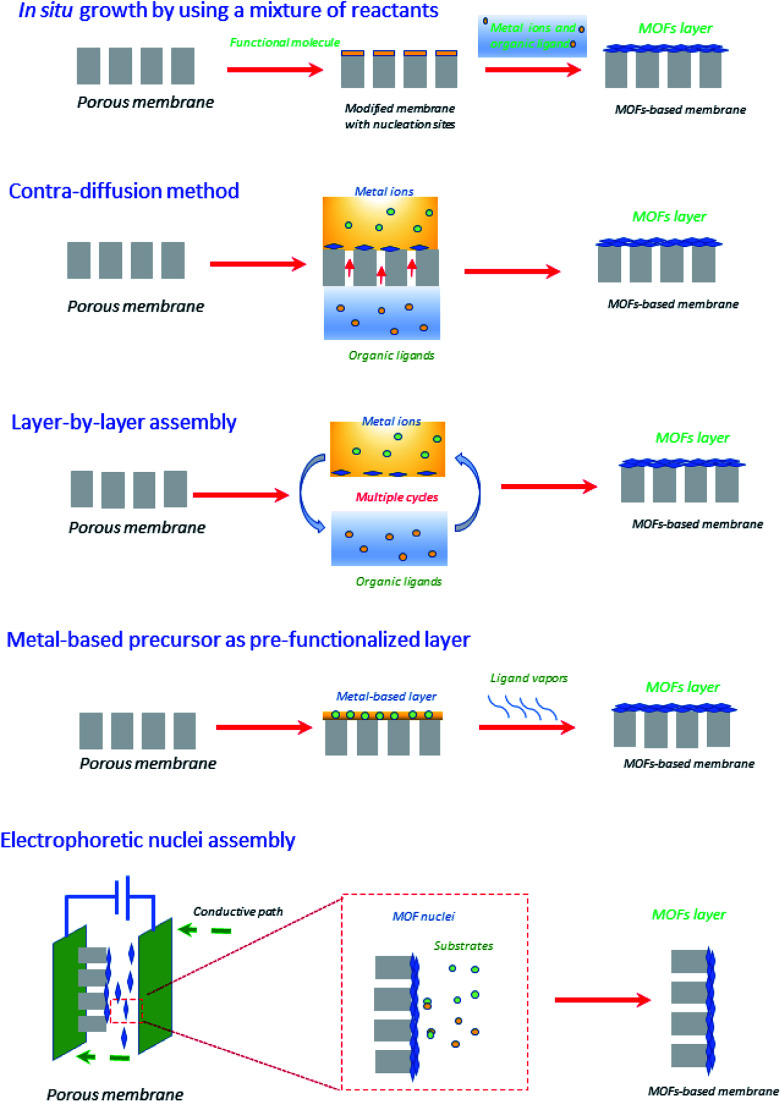
Graphical illustration of different strategies for creating ultrathin MOFs-based membranes.^44^

Very recently, Zhang *et al.*^44^ have reviewed emerging inorganic materials such as MOFs and COFs in the preparation of ultrathin membranes using advanced strategies such as *contra-diffusion method, in situ growth, layer-by-layer (LBL) assembly, laminated assembly of MOF/COF nanosheets, metal-based precursor as the pre-functionalized layer, and interface-assisted strategy.* To sum-up, the preparation of ultrathin membranes based on MOFs has been illustrated in Fig. 5. In particular, within the *in situ* growth protocol, the organic ligands and the metal ions are commonly mixed into one solution, in which the porous membrane support is immersed.^44,57^ At this point, the suitable selection of porous membrane supports should be taken into account in the MOF and even COF membranes due to the porous support surfaces (*e.g.*, the ones based on α-Al_2_O_3_ porous supports) must display specific affinity to promote the nucleation and thus MOF/COF formation. For instance, α-Al_2_O_3_ possesses oxygen motifs including strong coordination interaction with metal ions to enhance the nucleation densities, thus satisfying several rugged reaction conditions of COFs (for *e.g.*, polar solvents and high temperature). The drawbacks of *in situ* growth protocol are identified depending on the type of inorganic material, for *e.g.*, the use of MOFs is challenging in terms of scale-up, fabrication procedures, and final production costs. As such, MOFs have been also recognized by their poor hydrothermal and chemical stability and durability.^58^ However, given the diversity of chemical compositions, structures, and stabilities, proper selection of the MOF is of paramount importance in order to obtain the best matching with other components of the membrane. Regarding COFs, the successful construction of a wide variety of such crystalline COFs materials greatly depends on the reversible reactions. But, in return, it produces drastic disadvantages related to their hydrolytic/chemical stability, for *e.g.*, the presence of water during reversible COF formation may facilitate the backward reaction and thus the decomposition of COFs, making the use of COFs difficult in viable industrial approaches.^59^ To compensate in part for these drawbacks and as a counterpoint to what comes next regarding other membrane materials, MOFs and COFs can be prepared through alternative green methodologies that exclude solvents.^60,61^

In light of nucleation of MOFs, Knebel *et al.*^62^ developed a continuous ultrathin (*ca.* 200 nm) UiO-67 layer, which was designed by a high solvothermal process by mixing ZrCl_4_ and biphenyl-4,4′-dicarboxylic acid (BPDC) onto porous α-Al_2_O_3_. Such a membrane displayed acceptable separation factor values (in the range 5–29) towards different mixtures (*e.g.*, H_2_/CO_2_, H_2_/N_2_, H_2_/CH_4_, H_2_/C_2_H_6_, and H_2_/C_3_H_8_). Importantly, this MOF tends to provide thinner membranes when compared with other types of MOFs, such as HKUST-1 and ZIF-8, which gave a thickness of ∼20 μm using a similar substrate and synthetic protocol.^63,64^ In this way, it is clear that the type of MOF (including their primary elements of formation) and the porous support property is highly important for such an *in situ* growth strategy.

### 3.3 Atomic layer deposition (ALD)

**Fig. 6:**
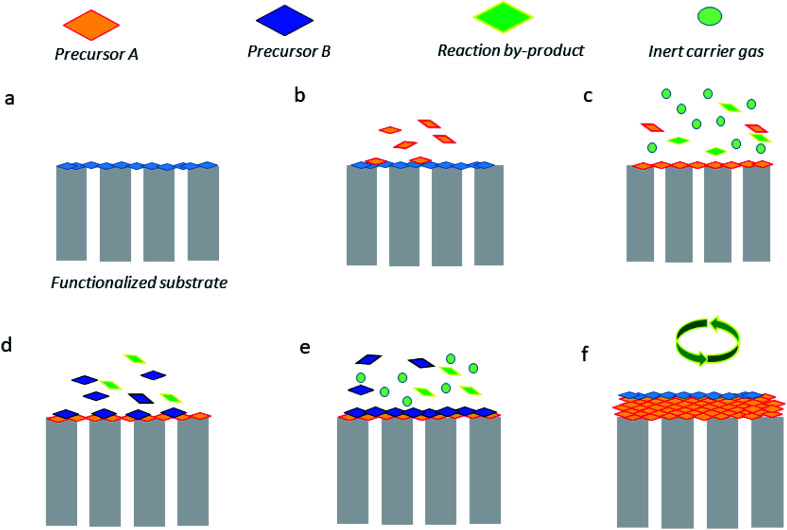
Graphical illustration of the most common stages of the ALD technique.^65^ (a) Functionalized support. (b) Precursor A reacts with the surface. (c) Purge of A and by-products. (d) Precursor B reacts with the surface. (e) Purge. (f) Repetition of (b–e) steps to achieve the target film thickness.

Atomic layer deposition (ALD) is likely one of the most used techniques in thin membrane preparation. ALD is recognized as a vapor phase technique that can produce thin films of plenty of materials. Such a technique offers multiple advantages including extraordinary conformality of high aspect ratio structures, thickness control at the angstrom range (0.1 nm), and tunable film composition.^65^Fig. 6 briefly illustrates how such a technique works, which can be described as follows. The substrate surface (or porous membrane support) should either be natural or be functionalized (a). Typically, the precursor A is pulsed and therefore reacts with the surface (b). When there is excess precursor and reaction by-products, they are purged with an inert carrier gas (*e.g.*, nitrogen or argon) (c). On the other hand, the precursor B is pulsed and reacts with the surface (d), and similarly, the remaining precursor and by-products are purged by using an inert gas (e). Finally, to reach the desired thickness, the stages (b–e) can be repeated. This technique gives the possibility of depositing several oxide precursor reactants by ALD (ZnO, TiO_2_, and Al_2_O_3_, among others) on diverse types of substrates (*e.g.*, ceramics and polymers) and thus convert them on different MOF materials. The drawbacks of ALD technique are the time required for chemical reactions, high quantity of wasted material, high energy consumption, and possible nanoparticle emissions.^66^ For instance, ZnO layers, which correspond to 5–70 nm size, have been deposited onto α-Al_2_O_3_.^67^ Subsequently, the conversion to ZIF-8 using a 2-methylimidazole–methanol solution under solvothermal conditions was achieved.^68^ The ZIF-8/ZnO/α-Al_2_O_3_ nanocomposite membranes were found to be H_2_ selective (permeance up to 1.6 × 10^−8^ mol· m^−2^ s^−1^ Pa^−1^) towards equimolar H_2_/CO_2_ and H_2_/CH_4_ gas mixtures with selectivity values of 7.8 and 12.5, respectively. In a different study, some authors also produced HKUST-1 thin films *via layer-by-layer* growth on ALD-coated fiber mats; the average thickness was about 117 nm. Surprisingly, this methodology was able to create a MOF monolayer per cycle, in which the thickness/cycle was close to 2.6 nm. It is important to mention that the authors did not evaluate the ability of the HKUST-1 membranes in separating gases but they should be potentially tested towards CO_2_/N_2_, CO_2_/CH_4_, O_2_/N_2_, and H_2_/CH_4_ separations^69,70^ and some other selective pervaporation separations.^71–73^ Importantly, ultrathin membranes prepared *via* ALD are capable of separating saturated vapors of organic solvents, for *e.g.*, Greil *et al.*^74^ evaluated the ability of ultrathin self-assembled block copolymer membrane (<50 nm) to separate acetone and ethyl acetate with the selectivity value of 7.

A similar approach was developed by Tran *et al.*,^75^ who performed the plasma-enhanced (by coupling with sol–gel method) ALD of titania (TiO_2_) on γ-Al_2_O_3_ porous support for its potential application as a H_2_-selective membrane. Using this methodology, after a number ALD cycles (280 cycles), a thin TiO_2_ layer with a thickness of ∼10 nm was obtained. Regarding gas permeation testing, the membrane presented a H_2_ permeance of approximately 12.5 × 10^−8^ mol m^−2^ s^−1^ Pa^−1^ and the H_2_/CO_2_ separation factor was about 5.8.

### 3.4 Chemical vapor deposition (CVD)

ALD is considered as a variant of *chemical vapor deposition* (*CVD*) in the deposition of thin films with highly precise thickness at the sub-monolayer level.^76^ The CVD technique has been recognized as advantageous compared to ALD due to ALD being a self-limiting layer-by-layer thin film deposition method. In particular, CVD employs the use of a substrate, which is exposed to one or more volatile precursors, reacting or decomposing on the substrate surface to produce the desired material. CVD usually guarantees excellent film quality and better control of film thickness.^77^ However, compared to ALD, the disadvantages of CVD are due to high temperatures required to decompose the precursor at the substrate surface, structurally defective material when synthetizing carbon nanotubes,^78^ possibility of producing gas by-products that are generally very toxic, uncontrollable thickness (when using graphene-based materials),^79^ and finally, similar to the other deposition techniques, CVD is also costly.^66^ Ultra-permeable poly[1-(trimethylsilyl)-1-propyne] (PTMSP) membranes have been proposed as supports to deposit metal–organic covalent networks, such as zinc(ii) *meso*-tetraphenylporphyrin (ZnTPP), *via* CVD.^80^ In such ZnTPP–PTMSP membranes with a thickness of 150 nm, the gas permeances of larger gas molecules (such as N_2_ and CH_4_) were significantly diminished, while high permeances of smaller gas molecules (H_2_) were obtained. Towards the preparation of thin ZIF-8 layers, Li *et al.*^81^ combined sol–gel coating with vapor deposition for solvent-/modification-free and precursor-/time-saving synthesis on the PVDF hollow fiber support. The resulting layers possessed a thickness of about 17 nm, which certainly provided high permeable properties for H_2_ (up to 215 × 10^−7^ mol m^−2^ s^−1^ Pa^−1^). When dealing with the selectivity, the values were of 3400, 1030, and 70 for H_2_/C_3_H_8_, CO_2_/C_3_H_8_, and C_3_H_6_/C_3_H_8_, respectively. With such impressive performances, the membranes overcame the Robeson (2008) limit for polymeric membranes. Furthermore, they also revealed a permeation cut-off between CO_2_ (*ca.* 0.33 nm) and O_2_ (*ca.* 0.346 nm) based on the crystallographic aperture of ZIF-8 (*ca.* 0.34 nm), providing selectivity values of 4.6, 7.3, 8.9, 14, and 940 for CO_2_/O_2_, CO_2_/N_2_, CO_2_/CH_4_, CO_2_/C_3_H_6_, and CO_2_/C_3_H_8_, respectively. At this point, such thin membranes are among the thinnest membranes reported in literature and therefore, the study addresses a viable method as an alternative for the scalable and controllable production of ultrathin gas separation membranes with unique and promising molecular sieving properties.

### 3.5 Electrophoretic deposition

Electrophoretic deposition has been used for quite some time to deposit charged colloids on to a substrate with the help of an electric field (*e.g.*, zeolite particles as seeds to growth a zeolite membrane)^82^ and has been recently demonstrated to synthesis sub-1 μm-thick high-performance MOF membranes.^83–85^ The flux of the colloids, *N*, is related to their concentration (*c*) and their velocity (*v*), as per eqn (3). *v* can be obtained from the mobility of colloid under the applied electric field, *E* (eqn (4)).

3 *N* = *cv*

4
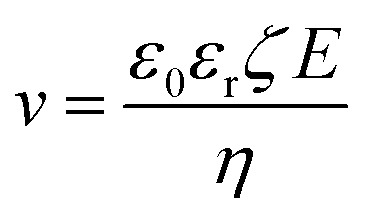
where *ε*_0_ and *ε*_r_ correspond to the permittivity of vacuum and dielectric constant of the medium, respectively. *ζ* corresponds to the zeta potential of charged particles and *η* refers to the viscosity of the medium.

The key advantage of using electrophoretic deposition for the synthesis of intergrown inorganic membranes is that one can carry out the synthesis directly from the precursor solution without the need for time-consuming separation of seed crystals. Further, this method can be optimized to deposit sub-100 nm-sized nuclei instead of large seed crystals. This has been demonstrated to promote intergrowth. Other advances include precise control on film deposition rate, high deposition rate, and prevention of agglomeration. The disadvantage of this approach is that the size and quality of the coating is limited by the electrode. For example, in the coating of reduced graphene oxide flakes, uncontrollable agglomeration of the flakes can be problematic.^79^ By applying an electric field in the early stage of crystallization, heterogeneous nucleation of the crystalline film can be precisely controlled. For example, He *et al.* could achieve a ZIF-8 nuclei (defined by ZIF-8 nanocrystals with particle size in the range of 10–20 nm) deposition rate of 30 nm min^−1^.^84^ The deposition of a 100 nm-thick nuclei film, followed by a short intergrowth step, gave rise to 500 nm-thick polycrystalline ZIF-8 films, which yielded extremely attractive performance in propene/propane separation (propylene permeance of 300 GPU and selectivity of 30). Interestingly, the synthesis of ZIF-8 membranes using fast-current-driven synthesis under an electric field can lead to sharpened molecular sieving performance, as shown recently by Zhou *et al.*, achieving propene/propane selectivity of greater than 300.^85^

### 3.6 Facile vacuum filtration

**Fig. 7:**
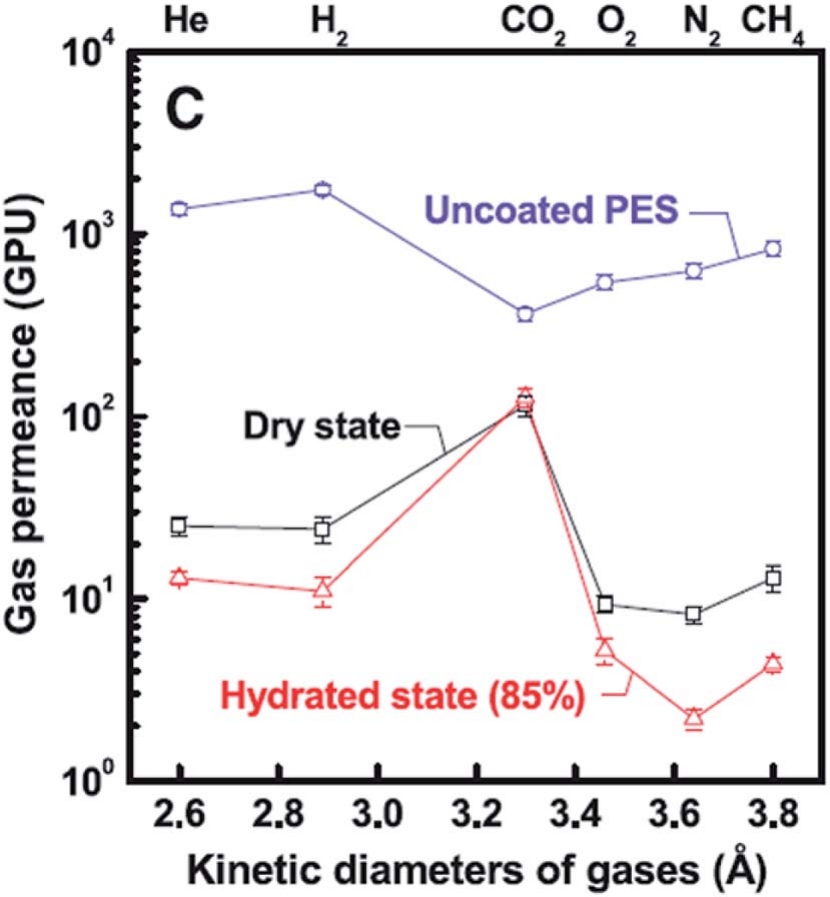
Gas transport behavior of ultrathin GO membranes prepared by Kim *et al.*^90^

Filtration has been recognized to be a useful protocol for assembling graphene and graphene oxide (GO)-based hybrid membranes due to the relatively facile procedure for creating controllable thickness and low-cost;^86^ however, the size and shape of the membranes are limited by the vacuum filtration device.^79^ These 2D materials, namely, graphene and GO, offer well-defined transport channels and atomic-thickness, giving extraordinary performance in gas separations (even for liquid separations).^87–89^ For instance, Kim *et al.*^90^ demonstrated that few- and several-layered graphene and GO sheets can be engineered to exhibit desired gas separation performance, for *e.g.*, the membranes were fabricated by contacting the support membrane surface to the air liquid interface of a GO solution, followed by spin-coating. Such a procedure allowed to obtain layered (3- to 10 nanometer) GO membranes, which displayed tunable gas transport behavior dependent on the degree of interlocking within the GO stacking structure, for *e.g.*, the CO_2_ permeability was ∼8500 Barrer, while the CO_2_/N_2_ selectivity was ∼20. Importantly, the authors also tested the gas permeance of these membranes (see Fig. 7), displaying gas permeance preference in the order CO_2_ > H_2_ ≥ He > CH_4_ > O_2_ > N_2_. Using the same 2D material, ultrathin molecular-sieving GO membranes for selective hydrogen separation were reported by Li *et al.*;^91^ in such a study, ultrathin GO membranes, with thickness approaching 1.8 nm, were prepared through a *facile vacuum filtration* process. These membranes displayed binary mixture separation selectivity values as high as 3400 and 900 for H_2_/CO_2_ and H_2_/N_2_ mixtures, respectively, through selective structural defects on GO. In particular, the authors found out that H_2_ and He permeances decreased exponentially as the membrane thickness was increased from 1.8 to 180 nm. Likewise, they speculated that the main gas transport pathway for these molecules was associated with selective structural defects within the GO flakes instead of *d*-spacing corresponding to the GO flakes.

Unlike Kim's approach (CO_2_/N_2_ = 20),^90^ Liu *et al.*^92^ obtained higher CO_2_/N_2_ selective (about 30) but less CO_2_ permeable (∼2100 GPU) membranes. In general, ZnTCPP nanosheets (∼3–4 nm) were synthesized *via* a surfactant-assisted technique (using polyvinylpyrrolidone as the surfactant) and subsequently deposited onto a flexible porous PAN support by vacuum filtration. This produced an ultrathin (∼25 nm) layer, which was used as a highly permeable gutter layer with reduced gas resistance in comparison with conventional PDMS.

Wang *et al.*^93^ reported ultrathin single-layered molybdenum disulfide (MoS_2_) onto AAO (anodic aluminum oxide, 200 nm pore size) membranes with controlled thicknesses using a simple filtration technique. For example, the thickness of the MoS_2_ membranes were in the range of 17–60 nm and the one possessing 17 nm thickness certainly displayed the best separation performance (*e.g.*, over 24 000 GPU for He and H_2_), which was governed by Knudsen gas transport mechanism (*i.e.*, no relevant molecular sieving properties were achieved). This occurred within the regular space between the MoS_2_ flakes and the larger stacking space in the MoS_2_ membrane (*e.g.*, 1.0 nm interlayer space). Finally, the highly permeable properties allowed to overcome the Robeson relationship even though the membranes had relatively low selectivity, *i.e.*, H_2_/CO_2_ = 3.4.

This technique of filtration can also be applied to suspensions of nanosheets of zeolites^94^ and MOFs^95,96^ obtained upon exfoliation of the corresponding crystals. In particular, the results achieved by Peng *et al.*^95^ are outstanding, with H_2_/CO_2_ separation selectivity of 291 at 120 °C together with the H_2_ permeance of 3760 GPU.

### 3.7 Interfacial polymerization (IP)

**Fig. 8:**
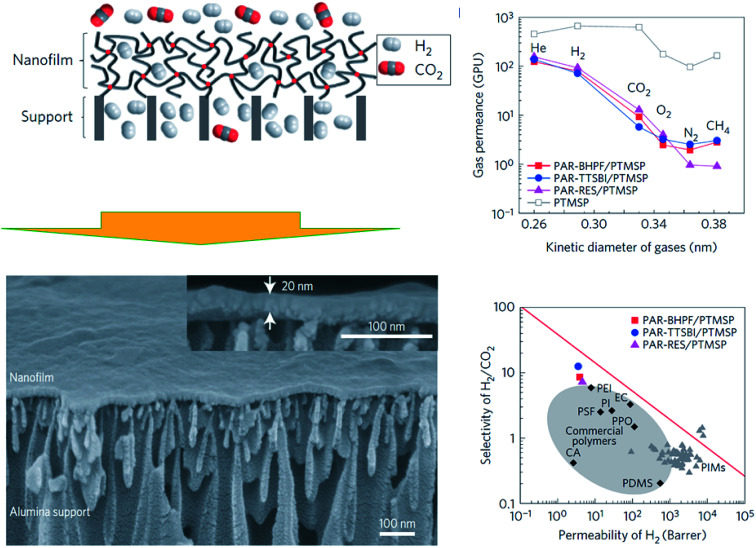
Interfacial polymerization of polyacrylate-based nanofilms and their gas separation performance. Adapted from Jimenez-Solomon *et al.*^99^

When dealing with the synthesis of polymer-based nanofilms, *i.e.*, the so-called thin film composite (TFC) or thin film nanocomposite (TFN) membranes, *interfacial polymerization* (*IP*) is a latent technique that provides highly crosslinked polymer films at nanoscale thickness. Typically, step-growth polymerization takes place at the interface between two immiscible phases (generally two liquids with a different monomer each), which results in a polymer that is constrained to the interface.^97^ In general, this procedure is not time-consuming (*i.e.*, fast reaction) but, unfortunately, it provides low yield, high cost of some its reactants (*e.g.*, acid chloride), and cannot run continuously. Also, there is a presence of remaining agents (*e.g.*, reactants and solvents).^98^ In Livingston's group,^99^ ultrathin cross-linked polyacrylate nanofilms supported on PTMSP (∼20 nm) were formed *in situ* by interfacial polymerization (see Fig. 8). Such nano-sized membranes possessed enhanced microporosity and higher interconnectivity of intermolecular network voids, which revealed defined molecular sieving features, *i.e.*, gas permeance decreased as He > H_2_ > CO_2_ > O_2_ > N_2_ ≈ CH_4_. Towards the overcoming of the so-called Robeson relationship, such nanomembranes clearly showed higher selectivity in comparison with typical polymer membranes with similar H_2_ permeability, positioning them near the trade-off (see Fig. 8). In any event, this performance has been recently overcome with analogous polyamide TFC membranes prepared on asymmetric polysulfone^35^ (H_2_/CO_2_ selectivity of 50 at 140 °C with a H_2_ permeance of 350 GPU) and polyimide^27^ (328 GPU of H_2_ and a H_2_/CO_2_ selectivity of 18.1 at 180 °C) supports.

Yuan *et al.*^100^ carried out IP using *N*-methyldiethanolamine (MEDA) and trimesoyl chloride (TMC) on crosslinked poly-dimethylsiloxane (PDMS) coated polysulfone support membrane. In general, the thickness of the PDMS skin-layer (between 49–308 nm) varied as a function of TMC content (from 0.0100 to 0.0750 mol L^−1^). In other words, TMC concentration governs the thickness of the membrane skin layer, in which higher TMC concentration promoted the formation of a more crosslinked membrane. In terms of permeance, higher TMC concentration (dealing with higher thickness) indeed contributed to less CO_2_ and N_2_ permeance values,^100^ for *e.g.*, the highest CO_2_ (up to 3000 GPU) and N_2_ permeances (up to 90 GPU) were obtained at the lowest TMC content (0.0100 mol L^−1^). In particular, within the IP technique, the monomers in both aqueous and organic phases are crucial in determining the thickness of the skin layer of thin film composite membranes.^101^ Very recently, Yu *et al.*^102^ manufactured ultrathin microporous polyarylate membranes (thickness between 25–75 nm) *via* IP of 5,5,6,6-tetrahydroxy-3,3,3,3-tetramethylspirobisindane (TTSBI) and TMC. The membranes exhibited CO_2_ permeance in the range 100–2115 GPU with CO_2_/N_2_ selectivity of 45–21. Herein, a specific membrane preparation parameter, such as the pH, has been identified as important towards membrane thickness. For example, the authors stated that the thickness of the selective layer gradually decreases with increasing pH value. This is due to the fact that the number of reactive groups (*i.e.*, –ONa) of TTSBI could differ under different pH values, which is obviously crucial to the IP method and membrane structure.^103^ Therefore, the pH value could remarkably affect the IP through hydrolysis of both TTSBI and TMC.

Two final remarks dealing with this technique are that: (i) filler nanoparticles can be incorporated during the IP process by maintaining the thickness of the membrane skin layer to an attractive value of *ca.* 100 nm (ref. 27) and is able to be operated up to 250 °C with H_2_/CO_2_ selectivity of 14.6 and a H_2_ permeance higher than 600 GPU; (ii) it can be applied to other polymer systems different from the typical polyamides used in the beginning of the development of the TFC membranes,^34^ as recently demonstrated by Shan *et al.*^37^ with the preparation of benzimidazole-linked polymer (BILPs) membranes with H_2_/CO_2_ selectivity up to 40 (at 24 GPU H_2_ permeance), high pressure resistance, and long-term stability (800 h in the presence of moisture).

### 3.8 Langmuir–Blodgett method

**Fig. 9:**
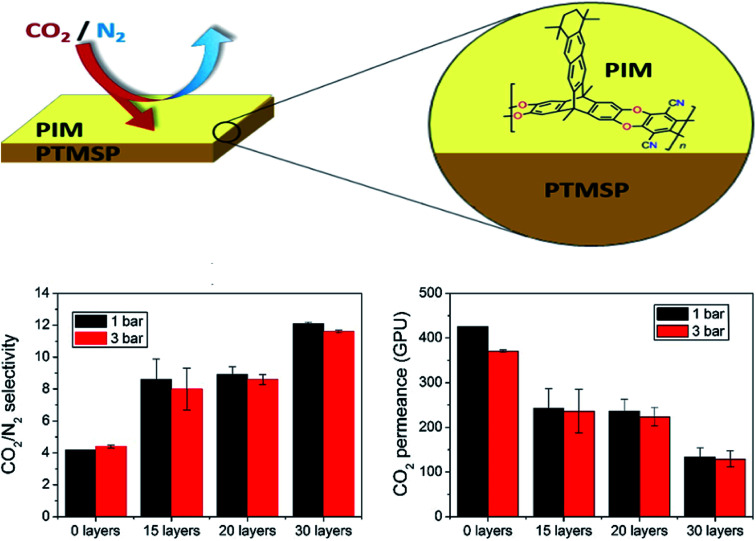
Separation performance of ultrathin PIM-PTMSP membranes as a function of number of PIM layers. Adapted from Benito *et al.*^110^

Langmuir–Blodgett (LB) is a technique generally used for the deposition of polymer-based monolayers on top of different types of membrane supports.^104^ Its disadvantages comprise equipment required for material processing, substrate size, film topology, and stability;^105^ however, the slow diffusion of substrates over the film and compressed floating films are typically obtained that are about one-fifth of the area of the trough, which restricts the quantity of the material that can be deposited onto a substrate in one batch. These features have, in fact, limited the commercial application of the LB technique.^106^ In general, several LB ultrathin membranes have been deposited onto PTMSP for enhancing the H_2_/CO_2_ and CO_2_/N_2_ separation. In this way, polymers of intrinsic microporosity (PIMs), as new kind of polymers with impressive gas separation performance (*e.g.*, PCO_2_ > 1000 Barrer and PCO_2_/PN_2_ ∼ 20),^107,108^ have been used for the manufacture of ultrathin membranes thick monolayers supported on PTMSP, which displayed CO_2_ permeance up to 7 times higher than that of dense pristine PIM membranes using only 0.04% of the mass of PIM (PIM-EA-TB(H_2_)), without a significant decrease in CO_2_/N_2_ selectivity.^29^ It is important to note that when the transference of the monolayer was horizontal, as was the case here, instead of vertical, the name of the technique is Langmuir–Schaefer (LS).^109^ In the same line, the authors deposited monolayers of the 2D polymer PIM-TMN-Trip onto PTMSP,^110^ displaying a performance which varied as a function of the number of layers on the selective film, as shown in Fig. 9, where the highest CO_2_/N_2_ selectivity (>10) was obtained in membranes having 30 PIM layers, whereas the maximum CO_2_ permeance (>400 GPU) was obtained for less number of PIM monolayers. Compared to the study by Benito *et al.*,^111^ it is likely that the polyelectrolyte multilayers on PTMSP membranes (thickness 20–32 nm) proposed by Lin *et al.*^112^ are closer to the empirical “upper-bound”. Certainly, these breakthroughs provide clear insights that the smart design of new types of membranes, together with emerging membrane preparation protocols, may favor to break the performance trade-off limitations of existing materials. Here, as a main drawback, the manufacture of ultrathin membranes *via* LB technique generally results in membranes with poor mechanical properties, which leads to cracks or application of macroporous support layers that are tough to cover by LB film without defects.^104^ Finally, LS/LB may allow controlled filler positioning in a mixed matrix membrane, as demonstrated with MIL-101(Cr) based TFN membranes for nanofiltration.^113^

### 3.9 Other emerging preparation protocols for the fabrication of ultrathin membranes

Recently, some other methods have been adopted in the preparation of ultrathin membranes towards successful gas separation. It is well known that selective layers, less than 100 nm thick, are highly desired for maximizing/optimizing the permeance of gas separation membranes for high energy efficiency. An extreme example of ultrathin selective layer is when the selective layer is only an atom thick, for *e.g.*, in the case of single-layer graphene membranes.^114,115^ Since the discovery of graphene in 2004, rapid advances in this field have propelled the synthesis of single-layer graphene in a scalable way.^116,117^ Further advances have led to the synthesis of large-area graphene membranes, made possible by mechanically reinforcing the graphene layer. For example, Huang *et al.*^118^ reinforced the graphene layer by a nanoporous carbon film and could synthesize a 1 mm^2^ sized single-layer graphene film with H_2_/CH_4_ selectivity of 6–25 and H_2_ permeance of 100–1000 GPU with a low porosity of 0.03%. Very recently, by reinforcing graphene by a carbon nanotube mesh, large-area graphene membrane could be prepared.^119^ This could also allow the fabrication of a tubular membrane module from single-layer graphene membrane, which displayed salt rejection between 85.2–93.4% and water permeance of 97.7 L m^−2^ h^−1^ bar^−1^. The key to obtaining high-performance membrane is to etch the otherwise impermeable graphene lattice in a controlled way. A proof-of-principle study was reported by Koenig *et al.*,^120^ where by etching micromechanically exfoliated pristine graphene in UV/ozone, CO_2_/CH_4_ and CO_2_/N_2_ selectivities exceeding 1000 were achieved from pressurized graphene microbubbles. Further progress in controlled and tunable etching of CVD-derived large-area graphene has led to angstrom resolution in molecular differentiation with H_2_/C_3_H_8_ selectivity exceeding 200 and H_2_ permeance up to 6000 GPU.^121^ Single-layer graphene allows for guest–host chemistry and can be an ideal matrix to form hybrid membranes. For example, using molecular dynamics simulations, Tian *et al.*^122^ recently showed that by decorating graphene nanopores with ionic-liquid, one can create ion-gated transport leading to an attractive CO_2_/CH_4_ selectivity of about 42 with a CO_2_ permeance of 10^5^ GPU. A similar concept was demonstrated by experiments, where He *et al.*^123^ functionalized single-layer graphene by CO_2_-selective polymer chains, achieving record high post-combustion carbon capture performance with CO_2_ permeance of 6180 GPU and CO_2_/N_2_ mixture separation factor over 22.5. Further developments in controlled etching of the graphene lattice, for example, by carbon gasification chemistry and functionalization of the lattice, is likely to allow a wide-range of industrially relevant gas separations from membranes based on single-layer graphene.

Pushing the thickness limit of conventional materials, Zhang *et al.*^124^ created a shear-aligned GO filled Pebax® 1657 hollow fiber membrane onto a porous polyvinylidene fluoride (PVDF) support by means of facile dip-coating technique. Particularly, the embedding of 0.1 wt% aligned GO laminates significantly enhanced the original Pebax® CO_2_ permeance properties from 220 up to 410 GPU, without compromising the selectivity properties towards CO_2_/N_2_ (∼45). This can be attributed to the defined GO *d*-spacing of ∼0.7 nm (intergallery distance of ∼0.35 nm), which is in the range of the kinetic diameters of the molecules (*e.g.*, CO_2_ = 0.33 nm and N_2_ = 0.36 nm). In addition to this, the composite membrane presented good operational stability and enhanced mechanical characteristics. It is important to note that a gutter layer based on PTMSP, as a highly permeable polymer, was applied to the PVDF support in order to skip the possible penetration of the coating layer into PVDF pores, favoring the creation of a true and effective ultrathin membrane. The use of a thin gutter layer was also proposed by Yoo *et al.*^125^ to prevent pore penetration in the selective layers. However, such a layer may provoke a decrease in the selectivity unless the gutter layer can be well designed. Based on this, Yoo *et al.*^125^ described a gutter material (*e.g.*, Teflon AF2400) that displayed six-folds higher CO_2_ permeance than PDMS (the most common gutter material). The membranes with ultrathin gutter (*e.g.*, 75 nm) and selective layers (*e.g.*, 70 nm) were tested for CO_2_/N_2_ separation, revealing a CO_2_ permeance >1455 GPU and selectivity of 68.1.

In a different approach, ethylenediamine (EDA)-functionalized GO flakes were used for the preparation of 28 nm EDA–GO membrane layer^126^ by deposition onto the inner surface of PES hollow fibers. Here, a vacuum-assisted coating method, consisting of seeding and coating steps, was applied. In terms of gas testing, such membranes displayed a high CO_2_ permeance of 660 GPU and a CO_2_/N_2_ selectivity of about 500. Moreover, it was reported that these functionalized membranes also presented selective water transport over gases and impressive water permeance >15 000 GPU.

A new route for the fast *in situ* growth of ZIF-8 membrane with the use of 2D graphitic carbon nitride (g-C_3_N_4_) was reported by Hou *et al.*^127^ In practice, 2-methylimidazole was used as a ligand solution for subsequent spin coating. The support (porous anodic aluminum oxide) was covered with Zn^2+^/g-C_3_N_4_ nanosheets and the ligand solution *via* cyclical spin coating. The obtained membranes displayed a thickness of about 200 nm that can be considered as thin, which is a result of the inhibition effect of the 2D nanosheets to avoid the growth of larger ZIF-8 particles.^127^ For instance, Table 2 enlists some other membranes based on ZIF-8 obtained using other methodologies and conditions, where it can be seen that spin coating meets the requirements of preparing ultrathin membranes. Similar to the study by Hou *et al.*,^127^ ultrathin ZIF-8 membranes with a thickness of about 200 nm were synthesized *via* chemical vapor modification of surface chemistry and nanopores of an asymmetric bromomethylated poly(2,6-dimethyl-1,4-phenylene oxide) (BPPO) substrate.^128^ Such membranes showed excellent H_2_ permeance (2.05 × 10^−6^ mol m^−2^ s^−1^ Pa^−1^) with acceptable H_2_/N_2_ and H_2_/CO_2_ selectivity values (9.7 and 12.8, respectively). For different types of separation (*i.e.*, propylene/propane), relatively thicker ZIF-8 hollow fiber membranes (thickness ∼800 nm) were designed by Joo *et al.*^129^ Basically, ZIF-8 membranes were supported on porous Matrimid® polymer hollow fibers by means of microwave-assisted seeding and microfluidic secondary growth. The authors packed densely ZIF-8 layers on hollow fibers under microwave heating. The ZIF-8 layers were then secondarily grown into well-intergrown ZIF-8 membranes under continuous flow of the growth solution. Likewise, the membranes exhibited a propylene/propane separation factor of ∼46 and propylene permeance of ∼55 GPU (permeability ∼ 49.4 Barrer). According to the authors' insights, these membranes are commercially attractive as they can overcome the upper bound of this separation and therefore, are located in the desired region. Besides, microfluidics can be used to access the interior of a hollow fiber support for proper synthesis of the active membrane material^130^ and to produce bilayered MOF membranes or to functionalize previously prepared MOF membranes by sequential pumping of the needed reactants.^131^

**Table 2:** Thin ZIF-8 membranes obtained with different techniques and synthetic conditions

Membrane	Technique	Time (h)	Temperature (°C)	Thickness (nm)	Reference
ZIF-8/g-C_3_N_4_	Spin coating	0.5	25	200	127
ZIF-8/PTSC	Counter-diffusion	48	25	620	132
ZIF-8/GO	Counter-diffusion	6	25	100	133
ZIF-8–MBPPO	Immersion	16	25	200	128
ZIF-8	Counter-diffusion	4	120	1500	134
ZIF-8@BPPO–EDA	Counter-diffusion	2	25	2000	135
ZIF-8	Secondary growth with seeding	6	30	2200	136

Nowadays, carbon molecular sieves (CMS) have emerged as highly promising membrane materials. CMS are typically have disordered packing of aromatic carbon strands, which are derived from the pyrolysis of a polymeric precursor.^137^ Such materials offer the possibility of being synthetized with a narrow pore-size-distribution, producing attractive sieving performances. Specific pores have been reported with a sub-angstrom resolution, displaying excellent molecular differentiation towards C_2_H_4_/C_2_H_6_ (ref. 138) and N_2_/CH_4_ (ref. 139) separations. In the framework of ultrathin CMS membranes, Huang *et al.*^140^ reported two fabrication ways, namely, transfer and masking techniques, allowing the preparation of CMS films of the order of 100 nm, providing attractive gas-sieving performances with H_2_ permeance reaching up to 3060 GPU and selectivity values between 18 and 24 for H_2_/CH_4_.

Highly permeable and oriented pseudozeolite aluminophosphate AlPO-18 membranes were prepared on the inner surface of tubular asymmetric alumina supports using directly synthesized nanosheets.^141^ In principle, AlPO-18 presented a thin sheet-like and hexagonal-prism morphology with a length of approximately 600 nm and thickness of only 30 nm. However, after the membrane preparation procedure, the coated layer grew into a continuous AlPO-18 layer and a thickness of approximately 1 μm was obtained. Regardless of the thicker layer of the membrane, the membranes gave a CO_2_ permeance as high as 3.6 × 10^−6^ mol m^−2^ s^−1^ Pa^−1^ (∼10 500 GPU), while the CO_2_/CH_4_ selectivity was about 91.5 at equimolar CO_2_/CH_4_ mixture.

PDMS/copper hydroxide nanofibers/(polyacrylonitrile) PAN ultrathin membrane layers (≈100 nm) were evaluated for CO_2_ capture applications (*e.g.*, CO_2_/N_2_ separation).^142^ Such composite layers were appropriately designed using generic interface-decoration-layer strategy. This generally involves molecular-scale organic–inorganic hybridization in the selective layer to obtain a high-performance ultrathin film composite. Such a membrane provided a 2.5-fold increase in the gas permeance up to 2860 GPU. Interestingly, the organic part of the composite material contributed to facilitate CO_2_-selective adsorption (*e.g.*, CO_2_/N_2_ selectivity of 28.2); on the other hand, the inorganic part helped to maintain a robust membrane structure.^142^ Therefore, a remarkable enhancement in the selective properties towards CO_2_ was obtained based on the synergistic effect of merging both inorganic and organic membranes.

Using atom transfer radical polymerization, Kim *et al.*^143^ developed ultrathin MMMs by merging bio-inspired iron(iii)–dopamine nanoparticles into a cross-linked selective layer (∼45 nm) of PEG macrocross-linker. Such a MMM selective layer was deposited on the top of the porous PAN substrate previously treated with a PDMS gutter layer. The CO_2_ permeance was about 1200 GPU, whereas the CO_2_/N_2_ selectivity was over 35. In such a study, the authors evaluated the performance of membranes in the presence of common gas contaminants, revealing that the CO_2_/N_2_ selective properties were still maintained over 4 months, while the CO_2_ permeance experienced a slight increase, which could be associated with the dissociation of coordinate bond between Fe^3+^ and dopamine in dry conditions.^144^ This might reduce the volume occupied by the nanoparticles, allowing the increase in the free volume and therefore, the enhanced CO_2_ and N_2_ permeances.

## 4. Concluding remarks and future trends

Even if the idea of ultrathin membrane is obvious (as the membrane thickness decreases both the membrane cost and permeance), there have been important technical limitations in realizing them over the past years. Throughout this study, we have reviewed the most applied and new techniques used by the research community aimed at the fabrication of ultrathin membranes for a synergic gas separation performance. Herein, we have identified that the most desired membranes are those that possess a thickness less than 100 nm. Nevertheless, according to the polymer and inorganic and hybrid materials, in addition to their design, relatively thicker membranes (let us say with thicknesses of selective layers below *ca.* 1 μm) may meet good separation performance and robustness as well. By reviewing the literature, it has been found that specific ultrathin preparation techniques (*e.g.*, *chemical vapor deposition and facile vacuum filtration*) are able to provide extremely thin membranes (*ca.* 17 nm) with highly permeable features (H_2_ permeance and good selectivities towards H_2_/CO_2_), which mostly surpass the Robeson trade-off. In fact, these values reveal that some procedures, even if highly promising, still need improvement to meet both high permeance and selectivity. In addition, three important suggestions for future research in the field can be noted: (i) evaluate the performance of thin membranes in long term operation. This could be useful in realizing the real potentiality of such a concept of membranes for real application and also, if there exists a limitation for the membrane stability as a function of membrane thickness for the same active material. (ii) The research community should start the testing of those membranes using complex gas mixtures that may contain real contaminants or some other organic components, as well as simulated close operating conditions. (iii) Towards successful industrialization, the fabrication of large-area membranes is also needed. However, the manufacture of membrane modules is still quite challenging since large-scale membranes with defect-free structures are difficult to obtain, which obligates resorting to healing techniques usually based on coating with elastomers such as PDMS.^145^ In addition to this, it is important to note that most of the approaches based on ultrathin membranes have been mainly focused on the preparation of flat-sheet membranes; therefore, if we seek commercialization and industrial consolidation, hollow fiber membrane modules are more attractive since they display less fouling and larger effective surface area, which can be translated to high productivities. Thereby, hollow fiber membranes seem to be the most feasible configurations and should be further explored, considering the high separation performance of some hollow fiber concepts, such as PIM-1/Matrimid,^146^ crosslinked PDMS,^147,148^ poly trimethyl phenylene ether sulfone,^149^ PIM/PDMS/PAN multi-layer composites,^150^ and those based on the crystallization of continuous MOF layers on these kind of supports^130,151^ and on the use of MOF based mixed matrix membranes.^152^ Several nano-materials have been tested in the preparation of thin layers; it is quite possible that 2D materials and COFs will be widely explored in the field. While the progress in applications of ultrathin-MOF membranes finds its main bottleneck due to the structural, chemical, and hydrothermal (acidic/basic) stability of the ligand–metal bonds, they are the dominant challenges to be faced in industrial applications. Moreover, it is time to explore new polymeric materials (*e.g.*, Teflon AF2400)^125^ as alternatives of traditional gutter layers (*e.g.*, PTMSP) onto porous substrates, which attempts the prevention of the coating layer into the pores of the substrates. Finally, it seems logic that particles of nanoporous materials with a high aspect ratio, *e.g.*, nanosheets, should be available with proper stability to create ultrathin membranes claimed in this review, particularly as mixed matrix membranes. In the particular case of MOFs (but this applies to all the materials described here), these nanosheets can be prepared by either exfoliation top-down approach^95,96^ or direct synthesis bottom-up approach.^153,154^ In any event, nanoporous nanosheets will most likely be the building blocks in creating a new generation of ultimate gas selective membranes.

To date, ALD technique has been likely the most used and explored technique since it can overcome many of the main drawbacks of conventional deposition techniques and it can be even be used to coat particles within ultrathin layers. Moreover, it allows to control the thickness (in the angstrom range, 0.1 nm) and tunable film composition. However, ALD is an analog of the CVD technique, which is most appropriate for binary compounds since a binary CVD reaction can easily be separated into two half-reactions.^79^ While other new techniques, such as electrophoretic deposition, have provided good insights into the fabrication of membranes but they still have limitations. It is clear that there is a growing trend in the development of ultrathin membranes in the future and the need to manufacture membranes in an eco-friendly manner (*e.g.*, electrophoretic deposition, electrochemical based techniques, and self-assembly). In summary, to select the right preparation protocol is a difficult task since it will depend on the type of membrane as well as its desired features, type of material (type of polymer and its physicochemical properties), type of inorganic phase (*e.g.*, size, shape, physicochemical properties, and stability of the nanomaterial), the cost incurred during the fabrication protocol, among others. Therefore, this paper has identified and provided to the readers the key advantages and drawbacks of each technique, providing a good guideline for the selection of a suitable ultrathin preparation technique according to their necessities.

## Nomenclature

AAO: Anodic aluminum oxide
AlPO: Aluminophosphate
ALD: Atomic layer deposition
ATRP: Atom transfer radical polymerization
BILP: Benzimidazole-linked polymer
BPPO: Bromomethylated poly(2,6-dimethyl-1,4-phenylene oxide)
CVD: Chemical vapor deposition
CMS: Carbon molecular sieve
COF: Covalent–organic framework
ED: Electrophoretic deposition
EDA: Ethylenediamine
*D*
: Diffusivity
GO: Graphene oxide
GPU: Gas permeation unit
GS: Gas separation
IP: Interfacial polymerization
LBL: Layer-by-layer
LB: Langmuir–Blodgett
LS: Langmuir–Schaefer
MOF: Metal–organic framework
MEDA: *N*-Methyl diethanolamine
MMM: Mixed matrix membrane
*P*
: Permeability
PAN: Polyacrylonitrile
PTMSP: Poly[1-(trimethylsilyl)-1-propyne]
PDMS: Poly-dimethylsiloxane
PES: Polyethersulfone
PEG: Polyethylene glycol
PVDF: Porous polyvinylidene fluoride
PIM: Polymer of intrinsic microporosity
*S*
: Solubility
TFC: Thin-film composite
TFN: Thin-film nanocomposite
TMC: Trimesoyl chloride
TTSBI: Tetramethylspirobisindane
ZnTPP: Zinc(ii) *meso*-tetraphenylporphyrin

## Conflicts of interest

The authors declare no conflict of interest.

## Supplementary Material
